# Reducing Risks in Petrochemical Plants Through the Integration of Existing and Emerging Gas Leak Detection Technologies

**DOI:** 10.3390/s25237197

**Published:** 2025-11-25

**Authors:** Joon Hyuk Lee, Sung Yoon Lim, Jae Joon Lee, Hongjin Shin, Youngsik Kim, Inkwon Kim

**Affiliations:** 1 Korean Fire Protection Association, Seoul 07328, Republic of Korea; jhlee@kfpa.or.kr; 2 Department of Fire Safety Engineering, Jeonju University, Jeonju-si 55069, Republic of Korea; 3 DL Chemical Inc., Yeosu-si 59611, Republic of Korea; hongjinshin@dlchemical.co.kr; 4 Stratio, Inc., Seongnam-si 13449, Republic of Korea; 5 SM Instruments, Inc., Daejeon 34109, Republic of Korea

**Keywords:** petrochemical plant, BREEZE, gas leak, arrangement of gas detectors, ultrasonic

## Abstract

Leakage of flammable gases and the resulting explosions in petrochemical plants remain latent risks, capable of occurring at any moment. Therefore, to address these worst-case scenarios within a virtual reality framework, we conducted simulations aimed at predicting and effectively responding to potential damages due to gas leakage. This study presents an analysis of the hazards that can lead to leaks and potential explosions in a petrochemical plant using the BREEZE Incident Analyst program. Rapid and accurate recognition of the risk associated with gas leaks, which can cause extensive damage and explosions, is of paramount importance. This study addresses two main aspects: the prediction of the consequences of gas leaks through simulations and the implementation of appropriate detection measures. Better, more efficient risk management and mitigation strategies were implemented by predicting gas leak paths using BREEZE. Using ultrasonic detection technology, detection was demonstrated to be possible in approximately one-third the time required by conventional detectors, and it is weather-insensitive. Simultaneously, considering plant characteristics such as utility configurations, we propose an additional method to prevent leaks from going undetected. This is achieved by integrating gas detection technology that combines both existing and new technologies.

## 1. Introduction

Accidents due to chemical leaks have become increasingly frequent in petrochemical complexes. These incidents have the potential to inflict severe damage to individuals and lead to large-scale accidents, including fires and explosions. If a leaked material is flammable, it can cause an explosion within a certain concentration range. An explosion may damage an area, the surrounding facilities, and human life. When an explosion occurs, it releases energy in the form of a blast overpressure that propagates through the atmosphere and an impact load through the ground. The degree of damage caused by an explosion depends on its explosive power, the situation at the point of the explosion, and the distance from the point of the explosion [1].

An explosion can be described as the rapid release of energy accompanied by a significant and sudden increase in gas volume, which often results in high temperatures and a considerable discharge of gases. Explosions also generate shock waves. A more precise definition of an explosion is a phenomenon in which the heat-generation rate of a combustible gas or liquid exceeds the normal rate. The speed of an explosion is usually around 0.3 m/s to 2 m/s. At the front of the detonation wave, the pressure is approximately 200–300 kbar (1 kbar is 1000 times the atmospheric pressure). The temperature of the gaseous products immediately behind it can reach 7000 °C [2].

It has been reported that explosion accidents may occur due to fire and leaks in oil refineries and petrochemical plants; therefore, it is necessary to review explosion safety standards so as to be better prepared to deal with such incidents [3,4]. These explosions are characterized by the release of significant explosive energy and the rapid expansion of gases [5,6,7]. The explosion process involves an intense oxidation-reduction reaction and represents a typical combustion process using pressure waves [7,8,9], which includes mechanisms that produce deflagration, detonation, and deflagration-to-detonation transitions of fuel-air mixtures for accidental large-scale vapor cloud explosions (VCEs) [10,11,12,13,14].

Violent explosions can occur because of the ignition of flammable gases or vapors, such as mixtures of air and hydrogen or hydrocarbons. Associated accidents have shown that extremely destructive explosions can occur owing to the ignition of large clouds containing hydrocarbon-air mixtures [14]. Incidents like the VPEs at Buncefield [15,16,17], Puerto Rico [18] and Jaipur [19,20] have altered our understanding of their potential burn intensity. Under defined atmospheric conditions and certain fuel-air mixtures, powerful and rapid combustion phenomena and explosions can occur in VCE.

The analysis program used in the BREEZE Incident Analyst software (version 2.0.0.9) used in this paper is the SLAB model, developed by the U.S. Department of Energy and Lawrence Livermore National Laboratory (LLNL), which simulates the atmospheric dispersion of denser-than-air gases. The SLAB model, developed at LLNL, simulates evaporation pools, horizontal and vertical jets, and burst discharges. The model averages the conservation equations of mass, momentum, energy, and species to distinguish between steady plumes (sustained discharges) and unsteady puffs (sudden discharges). The model quantifies these data by including gravitational diffusion, turbulent mixing, heat transfer, and droplet evaporation [21].

The wind rose data for the Yeosu region, selected for analysis in this paper, were obtained from the BRIDGE platform (https://bridge.kfpa.or.kr, accessed on 10 October 2025) of the Korea Fire Insurance Association (KFPA), which provides risk assessment data to the Korean insurance industry. The BRIDGE platform provides visualizations of annual data on wind direction from the Korea Meteorological Administration in the relevant region.

In this study, risk factors were identified by simulating and analyzing gas leak accidents in petrochemical plants. Processes related to C4, which is a major intermediate in petrochemical processes, were selected. Risk analysis for this process was not performed as much as for the other processes. Gas leakage during the hydrocarbon synthesis process was assumed based on accident cases and regulations. Depending on the accident scenario, the gas diffusion and overpressure effects were quantitatively evaluated through simulations. Based on the results derived from the simulation results, we present a method for detecting gas leaks in an area more efficiently and accurately. In addition, we present an appropriate method for combining new and existing technologies based on the influence of air currents.

## 2. Gas Leak Accident Cases

Most causes of gas leaks in petrochemical plants involve non-compliance with safety rules or facility defects. Among facility defects, process-line damage has been identified as the main cause of leakage, including failure, deterioration, and corrosion [22,23].

There have been accidents in which process piping leaked while being filled with steam. The cause of the accident was presumed to have been damage to the area around the cracked pipe weld. During this process, hydrocarbon gas leaked, mixed with oxygen from the air, and was exposed to sparks, leading to ignition. In addition, there is potential for ignition due to static electricity when the gas floats or shakes because of its low electrical conductivity. This series of factors can collectively contribute to the occurrence of explosions [24].

Table 1 lists the statistics of explosion accidents due to gas leaks in Korea; the data is taken from the National Fire Data System (NFDS, National Fire Agency) [23] of Korea’s National Fire Administration. Damage to life and property due to gas leaks (explosions) showed a decreasing trend from 2018 to 2021, but increased significantly in 2022. The number of accidents in 2022 was lower than the average of 165 from 2018 to 2021; however, the number of fatalities and property damage was higher. In particular, property damage in 2022 increased by approximately 10 times compared with that in 2021. It appears that this was due to a major accident that caused significant damage. Explosions caused by gas leaks statistically result in significant human and property damage.

## 3. Cause Analysis and Scenario Setup

### 3.1. Causes and Locations of Gas Leak

Accidents such as fires or explosions in petrochemical plants are caused by fluid leakage in pipes, valves, pumps, and vessels, and are primarily caused by human error and poor maintenance by workers. If the facility is outdated or maintenance is insufficient, leakage may occur because of a pinhole in the pipe or a poor joint connection. Among recent chemical spill accidents, physical factors have been shown to cause many leak accidents owing to failure, damage, deterioration, and corrosion [24].

Pipeline leaks in petrochemical plants can occur in many places due to various causes. From investigating and reviewing accident cases due to leaks in petrochemical plants, the most common areas are not only corroded parts of pipelines but also joints and fittings, which generally include flanges, valves, and seals [22,25,26,27].

These leaks typically result from a combination of factors, including mechanical failure, human error, and environmental conditions. Mechanical failures can be caused by the aging, stress, and wear of pipeline materials and components. Human errors can occur during the operation, maintenance, or installation of pipelines, such as improper sealing, incorrect operation of valves, and a lack of regular inspections. Environmental conditions such as temperature changes, ground movement, and corrosive soils can also cause pipeline failure [22,23].

Therefore, prevention and rapid detection are critical for managing these risks. Regular inspection and maintenance, proper personnel training, the use of corrosion-resistant materials, and leak detection techniques must be applied as appropriate. Among these, advanced leak detection technologies can play an important role in minimizing the impact of pipeline leaks at petrochemical plants.

In ASME B31.3, paragraph 345.3 (Preparation for Leak Test), factory operation starts after conducting a leak test for all joints and welded parts to prevent leakage during operation. It stipulates that gas leak tests should be conducted on structurally weak parts, such as welded parts [28].

Based on statistical facts, investigation reports, and regulations, gas leaks owing to weld failure were designated as the cause of explosions. Damage analysis similar to an actual leak was conducted by performing a simulation.

### 3.2. Gas Leak Scenario

Based on cases of gas leak accidents in petrochemical plants, a gas leak scenario in the hydrocarbon synthesis process, which is one of the major processes in the plant, was established. In the synthesis process, 1-butene is separated into liquid and gas phases through a gas stripper, and the gas phase 1-butene is transported through a pipe at the top of the stripper. By simulating a scenario in which gaseous 1-butene leaked, the gas diffusion range and effect of overpressure by the VCE were analyzed under various conditions. Based on the results of the field investigations and drawing reviews, simulations were performed based on the values presented in Table 2 in an environment similar to a process unit operating in a petrochemical plant.

## 4. Incident Analysis Due to Gas Leak

### 4.1. BREEZE Incident Analyst Program

In this study, simulations focusing on gas leaks and explosions were performed using the BREEZE Incident Analyst developed by Trinity Consultants. BREEZE Incident Analyst is a chemical accident damage prediction program that is widely used by the US EPA, Coast Guard, and many other industries. It is designed to quantitatively analyze the spread of combustible and toxic gases and the scope of the influence of fires and explosions based on various theories.

The SLAB model used in BREEZE is a computer model that simulates the atmospheric dispersion of denser-than-air emissions. A recent study was published that modeled chlorine gas diffusion in an industrial environment using BREEZE Incident Analyst software [29]. The model addresses ground-level evaporating pools, elevated horizontal jets, stacks, or elevated vertical jets, and instantaneous volume sources. The atmospheric dispersion of emissions is calculated by solving conservation equations for mass, momentum, energy, and chemical species. These conservation equations are spatially averaged, treating clouds as steady-state plumes, transient puffs, or a combination of the two, depending on the duration of the emission. The conservation equations inherently incorporate a mathematical description of the physical properties of heavy gas dispersion (e.g., gravitational diffusion, reduced turbulent mixing), as well as the processes of normal atmospheric advection and turbulent diffusion. The time-averaged concentration predicted by the SLAB model is influenced by various physical phenomena related to the dispersion equations, as well as the specific concentration averaging time.

### 4.2. Leak Material

1-Butene, one of the streams used in petrochemical processes, was selected. 1-Butene was contained in the C4 fraction recovered from the gas generated by cracking during petrochemical processes. This substance is used in various processes; however, few studies have been conducted on its dangers. Unsaturated aliphatic hydrocarbons, such as 1-butene, are generally much more reactive than alkanes. As a combustible gas, it can cause evaporative combustion and has a flash point of −79 °C. Its flammability limit in the air ranges between 1.6 and 9.3%, wherein explosions by ignition sources are possible. It can also act as an asphyxiant or a mild anesthetic at high concentrations, causing eye irritation [30].

However, in-depth research on the hazards of 1-butene has been largely lacking. 1-butene is classified as an extremely flammable gas on the MSDS and poses a risk of explosion when heated, making it extremely hazardous.

Because of its extremely low flash point of −80 °C, it is also classified as the most hazardous substance according to the hazardous material classification of domestic insurance companies. Furthermore, the precautionary measures on the MSDS clearly state that all ignition sources, including surrounding heat, sparks, flames, and high temperatures, must be eliminated.

Therefore, 1-butene, a substance used in petrochemical processes and known to pose risks but lacking prior research, was selected for simulation. Although there are some previous studies, including simulations, conducted on 1-butene, most of them are related to processes and reactions involving 1-butene [31,32,33]. Therefore, research on leakage of 1-butene alone is very limited to date, and it is believed that this could serve as a good reference for future research.

This substance is not only an explosion hazard but can also have harmful effects on the human body. However, in the case of leakage, elements harmful to the human body are generated at high concentrations. Therefore, early detection and response can help prevent the occurrence of these risks. In addition, even if the range of flammability limits in air is met, an explosion can be prevented if the leak source is quickly determined before ignition.

### 4.3. Parameter Setting

The meteorological conditions, considering the annual average temperature, humidity, and wind speed of the Yeosu Industrial Complex in 2022, provided by the Korea Meteorological Administration (KMA), were applied as parameters, as shown in Table 3.

**Figure 1:**
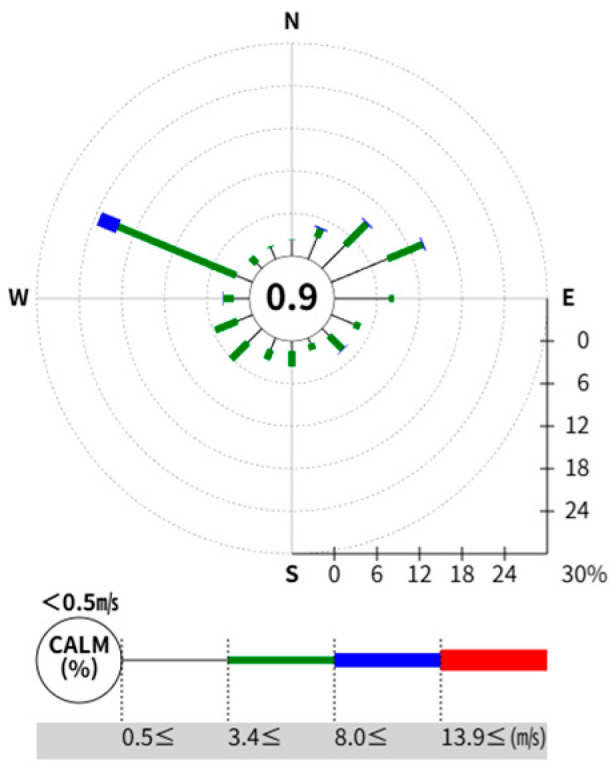
Wind rose data.

**Figure 2:**
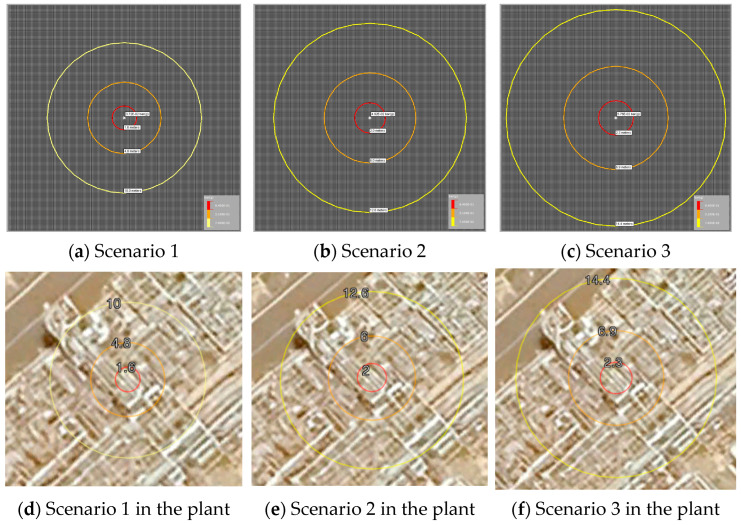
Results of displayed threat zone (explosion of 1−butene).

As shown in Figure 1, a wind rose provides a concise view of the general distribution of wind speed and direction at a particular location. As shown in Figure 2, a wind rose, represented as a circle, represents the frequency of wind blowing from a particular direction.

### 4.4. Analysis of Damage Impact Due to Explosion Overpressure

The range of influence of the explosion according to the retention state of the combustible mixture at the top of the gas stripper was analyzed. 1-Butene is a flammable gas that is heavier than air, and VCE may occur because of the accumulation of flammable mixtures within confined spaces created by facilities. Table 4 lists the analysis criteria based on explosion results.

**Figure 3:**
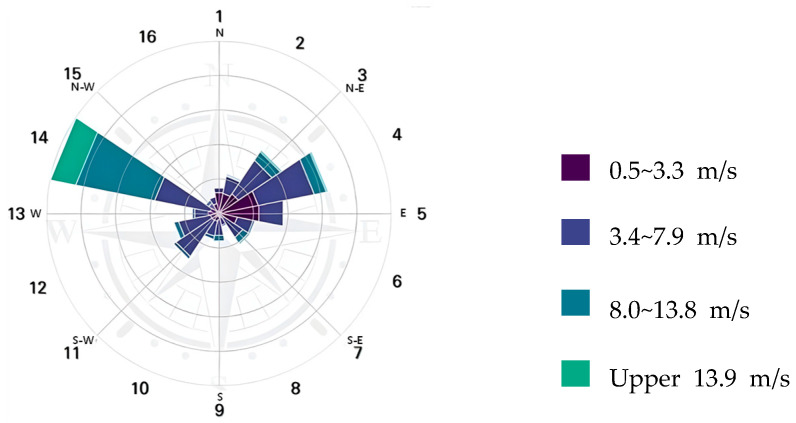
Wind direction and wind speed distribution in Yeosu Industrial Complex.

The confined volume (fuel-air) that could be generated was set based on the configuration of the equipment installed in the gas stripper. An explosive strength of 7 was applied based on the Kinsella guidelines [34], considering the intensity of the ignition source, obstacle, and degree of retention [35]. Based on the VCE analysis, it has been determined that under various conditions, the force generated by an explosion that can break windows can cause damage up to a distance of 10 m. Additionally, enough force that can cause equipment damage can destroy nearby objects within a distance of 1.6 m. The explosion in Scenario 3 was found to cause damage up to a distance of 14.4 m. The explosion ranges for each scenario are summarized in Table 5. The range of influence of the pressure is shown in Figure 3. Explosions in each scenario, particularly in areas with relatively dense process facilities, storage tanks, and control rooms, can easily damage the surroundings. Therefore, the early detection of gas leaks holds significant importance in plant processes.

### 4.5. Gas Diffusion Analysis and Detector Arrangement

#### 4.5.1. Analysis of Influence Range According to Gas Diffusion

**Figure 4:**
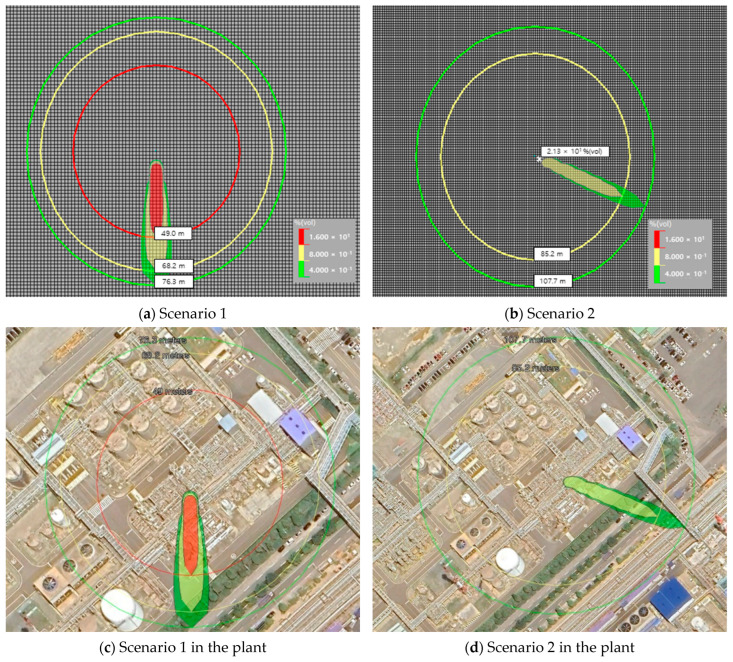
Results of the displayed threat zone (leakage of 1−butene).

In the gas diffusion model, we assumed a scenario in which the leakage of 1-butene occurred owing to the rupture of a 4 in pipe connected to the top of the gas stripper. In this scenario, the upper section of the gas stripper is situated in an outdoor open space, and the results can differ based on the influence of the wind speed and direction. Therefore, as shown in Figure 4, we simulated a scenario that considered the higher frequencies among the 16 wind directions at the point of analysis.

1-Butene is heavier than air, and its atmospheric diffusion effect was analyzed using the SLAB model. The SLAB model is a dense gas dispersion model used to estimate windward pollutant concentrations resulting from accidental chemical releases. The gas diffusion criterion was set based on the Lower Explosion Limit (LEL) of 1-butene (1.6%). In Korea, detectors are installed and managed according to the Technical Guidelines for Installation and Maintenance of Gas Leak Detection and Alarm (KOSHA GUIDE, P-166-2020) [36]. In the case of two or more alarm settings, the standard stipulates that the first (high) alarm should be set at 25% or less of the lower explosion limit, and the second (high-high) alarm should be set at 50% or less of the lower explosion limit. As shown in Table 6, the reach distance was predicted, with the influence range reaching 100%, 50%, and 25% of the LEL.

**Figure 5:**
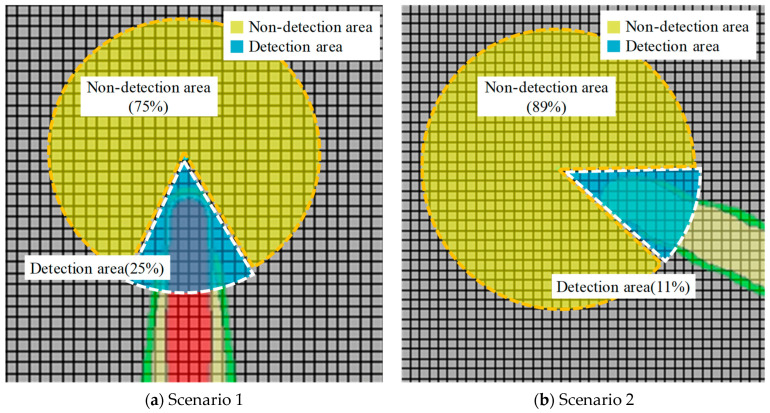
Probability of detection by gas leak scenario.

The type and concentration of the leaked gas depend on wind speed and direction. Table 7 summarizes the results of each scenario. As the wind speed increased, the diffusion distance increased; however, the gas leak area and concentration decreased. In Figure 5, the difference between the two scenarios can be intuitively recognized.

#### 4.5.2. Limitations of Existing Gas Leak Detectors

The results in Table 7 and Figure 5 show the diffusion range of the gas according to the wind direction and wind speed. Scenario 1 can detect up to 25% of the LEL within a radius of 76.3 m in a due southerly direction. In Scenario 2, detection was possible up to 25% of the LEL within a radius of 76.3 m in the southeast direction. However, as shown in the results of the two scenarios, it can be seen that the range of influence of gas diffusion spreads in a limited direction depending on the wind direction and speed. In addition, from comparing the results of scenarios 1 and 2, it can be seen that the higher the wind speed, the wider the diffusion range, but the lower the retained concentration.

**Figure 6:**
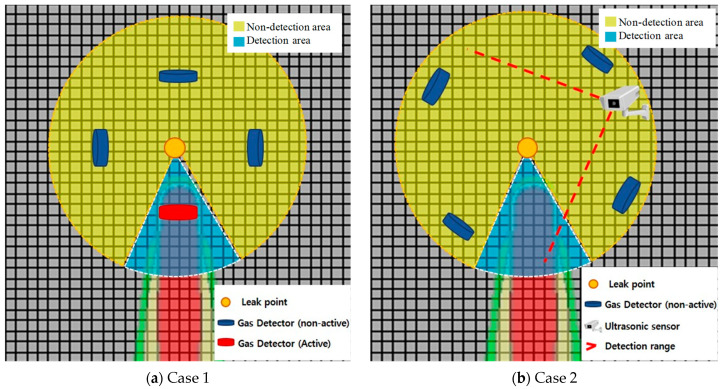
Probability of detection by arrangement of detectors in Scenario 1.

As shown in Figure 6, as a result of the scenario, the effective range of gas detection according to the wind direction was confirmed to be approximately 11 to 25%. To effectively detect leaked gas, it is possible to increase the detection probability by estimating a path, such as the diffusion direction and the moving distance of the gas, and appropriately arranging the gas detection unit; however, there are clear limitations. It is difficult to detect all gas leaks caused by air currents using the concentration measurement detectors installed in existing petrochemical plants. The gas stripper selected for the simulation included a gas leak source exposed to the atmosphere in the upper part of the outdoor area. Therefore, it is difficult to determine an appropriate concentration for detecting leaked gas owing to the influence of the atmospheric environment, such as wind speed and wind direction, and the probability of an initial detection failure is high. In the case of handling a large amount of flammable materials, such as in a petrochemical plant, large-scale damage, such as an explosion, may occur owing to failure to detect the leaked gas in the initial stage. Rapid detection of leaked gas is important in terms of risk management.

## 5. Minimizing Accidental Leak Damage

Through the explosion simulation in Section 4.4, it was inferred that enormous damage could be caused if a VCE occurs owing to a gas leak. Thus, if an explosive atmosphere can be detected before it is formed and an early response is possible, a disaster that causes enormous losses can be prevented. In Section 4.5, simulations are performed to show the gas diffusion due to airflow in the event of a gas leak. Based on this, the probability of detecting a gas leak early can be partially increased by arranging a gas leak detector to quickly and efficiently measure the gas. However, this is not the ultimate goal of this study.

**Figure 7:**
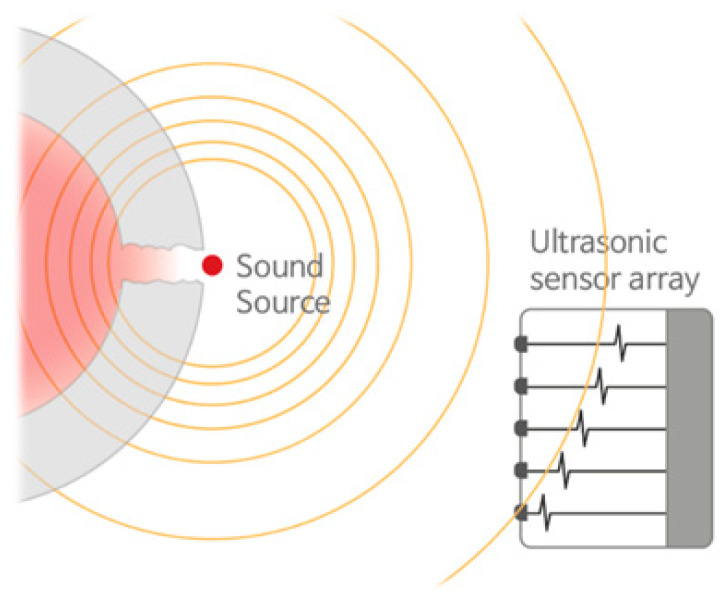
Generation of leakage signals and measurement using arrays [37].

Figure 7 shows whether detection was performed according to the arrangement of detectors in Scenario 1’s leakage, represented in Table 7 and Figure 5 and Figure 6. The detector arrangement was divided into two cases.

As shown in Figure 7, one detector was operated by airflow in Case 1. In Case 2, despite the placement of four detectors around the source of the leak, the airflow caused the gas to diffuse between the detectors and was not detected. Therefore, considering the frequency of airflow diffusion, it is reasonable to arrange the detectors as in Case 1. In addition, in Case 2, if an ultrasonic sensor is added, gas leaks can be detected by monitoring the area itself, regardless of the airflow. Unlike conventional concentration measurement methods, ultrasonic sensors can detect a specific area from a distance using space-sensing technology, such as CCTV. Therefore, if the technology is added to the existing detection system, the drawbacks of the existing gas leak detection can be supplemented.

### 5.1. Principles of Ultrasonic Sensors

**Figure 8:**
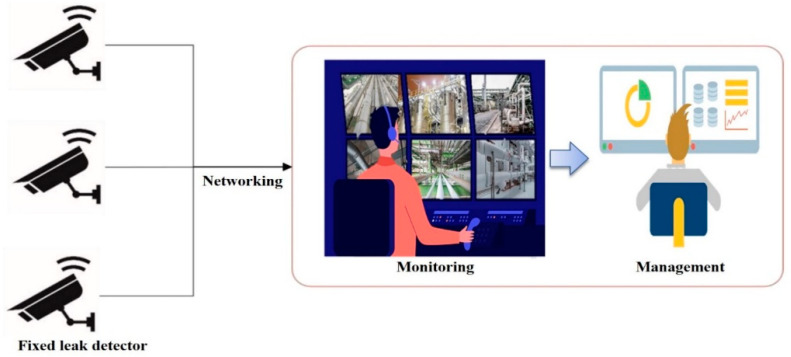
Schematic of gas leak monitoring.

The location of a leak in a petrochemical plant can be determined using ultrasonic detection technology as follows: If a gap or hole due to damage occurs in a pressure-filled pipe connection, as shown in Figure 8, air leaks owing to the pressure in the pipe, and a vortex of air forms at the leaked part. The signal generated by this vortex phenomenon appeared as an ultrasonic band that could not be heard by humans [38]. At this time, the ultrasonic signal generated by the leakage appeared to be more prominent than the other signals [37,39].

These signals propagate through the air and reach the microphone sensor. If there are several microphone sensors, signals arrive at each sensor at different times. When the signals are analyzed and processed, the location of the signal source can be determined [40]. Acoustic wave measurements can also be used outdoors, where wind blows and air spreads quickly. Generally, gas detectors installed in petrochemical plants to measure concentrations are difficult to operate in such environments. Additionally, ultrasonic measurements can be performed regardless of the type of air or chemical gas used. Ultrasonic gas leak sensors are increasingly used in petrochemical plants.

### 5.2. Applications of Ultrasonic Sensors

**Figure 9:**
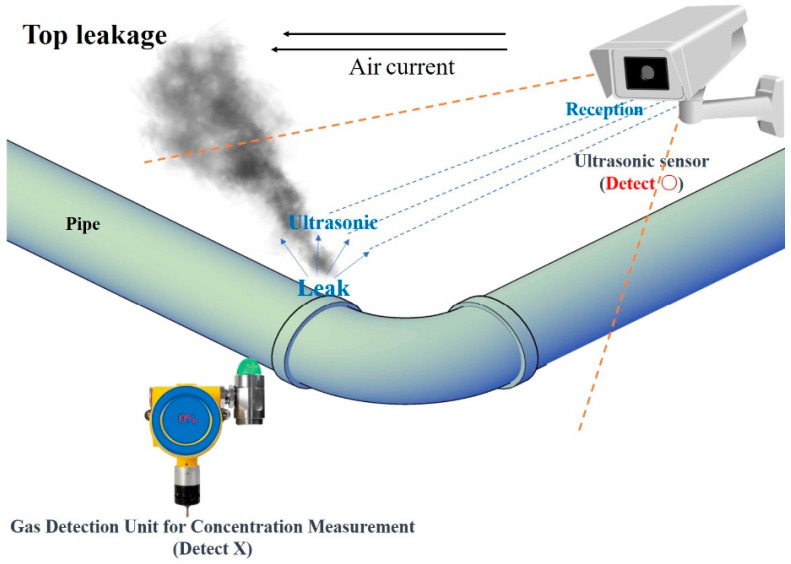
Cross-check method of gas leak detection caused by a failed elbow (top).

By employing ultrasonic waves in gas leak detectors, it is possible to monitor the ultrasonic waves received through a network, as shown in Figure 9. The results obtained from the ultrasonic microphone sensor array are transmitted to the server through the network. Online surveillance and monitoring are possible using the transmitted data. The transmitted results are displayed in real time on a dashboard and stored simultaneously, allowing users to manage their history [41]. Similarly to existing gas leak detection systems, ultrasonic sensors can be reviewed for deployment in the field by applying a network to existing portable ultrasonic measuring devices.

### 5.3. Combining Existing Gas Leak Detector and Ultrasonic Detection Technology

Petrochemical plants are expected to become safer by utilizing the proper arrangement of existing gas leak detectors through simulations and ultrasonic detection technology. Considering the environment of each zone, areas where gas leak detectors have difficulty measuring are reinforced with ultrasonic detection technology. Conversely, even if ultrasonic waves are generated in the case of a gas leak, a blind spot may occur in which ultrasonic waves are not transmitted to the sensor array of the ultrasonic detection facility because of obstacles. This part was supplemented as much as possible by using an existing detection system. In addition, because the ultrasonic detection technology developed thus far recognizes other ultrasonic waves generated in the vicinity and may be misrecognized, it is important to determine the exact leak source by checking with existing detectors in a cross-wise manner.

Joint damage may occur due to thermal expansion and contraction at the bent portion of the pipe. Leakage is likely to occur in this part, and in the case of joint leakage in such a curved pipe, a countermeasure must be implemented by the combined arrangement of an existing detection system and an ultrasonic detection system.

**Figure 10:**
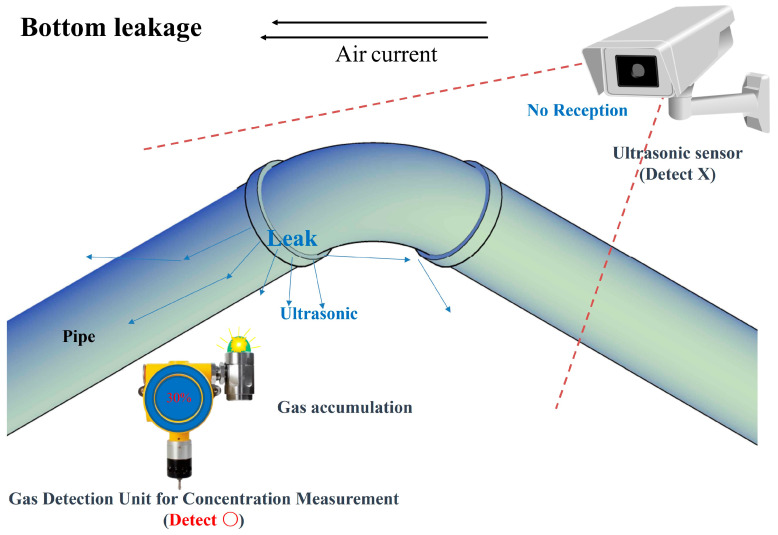
Cross-check method of gas leak detection caused by a failed elbow (bottom).

Assuming a leak in the bent pipe, the layouts of the ultrasonic sensor and the existing concentration measurement method gas detector based on the leak site were proposed, as shown in Figure 9 and Figure 10, respectively.

Figure 9 and Figure 10 show examples of the application of cross-detection technology for the two technologies based on the gas leak area and airflow. In addition, this configuration complements each detector’s weaknesses by using two sensing technologies. The gas detection unit of the concentration measurement method was efficiently arranged by predicting gas diffusion according to the air flow described in the previous section. The ultrasonic sensor should be arranged without blind spots as much as possible in preparation for cases in which it is difficult to measure the gas detection unit of the concentration measurement method.

In Figure 9, it is assumed that a gas leak occurred at the upper section of the joint of the bent pipe, in a scenario where the surrounding airflow is moving towards the left. In this case, the leaked gas diffused into the surroundings without accumulating in the direction of the gas detector. However, leaks can be identified using an ultrasonic sensor installed on top.

Similarly to Figure 9, Figure 10 assumes a situation in which leakage occurs at the joint of the bent pipe and the airflow is directed to the left. The only difference was in the location of the leakage, which was at the lower end of the junction. In this case, the ultrasonic waves generated from the leaking part are blocked by obstacles (piping or various facilities and structures existing in the petrochemical plant) and cannot be measured using an ultrasonic sensor. However, it was possible to detect the accumulated concentration in the direction of the concentration-measuring gas detector. This is the opposite of the detection situation shown in Figure 9, in which case the blind spot of the ultrasonic sensor can be compensated for by the detector of the concentration measurement method.

**Figure 11:**
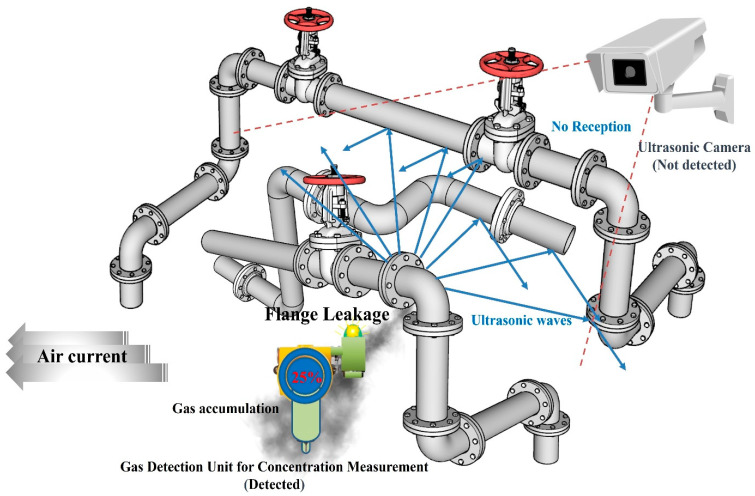
Blind spots in ultrasonic sensors due to obstacles.

Figure 11 shows the gas leak detection situation under more complex piping configurations. In this case, the air flow was directed toward the concentration-measuring gas detector. It was possible to detect gas leaks by accumulating near the concentration-measuring gas detector. The ultrasonic sensor could not receive the ultrasonic waves generated from the leak because they were blocked by structures such as surrounding pipes.

**Figure 12:**
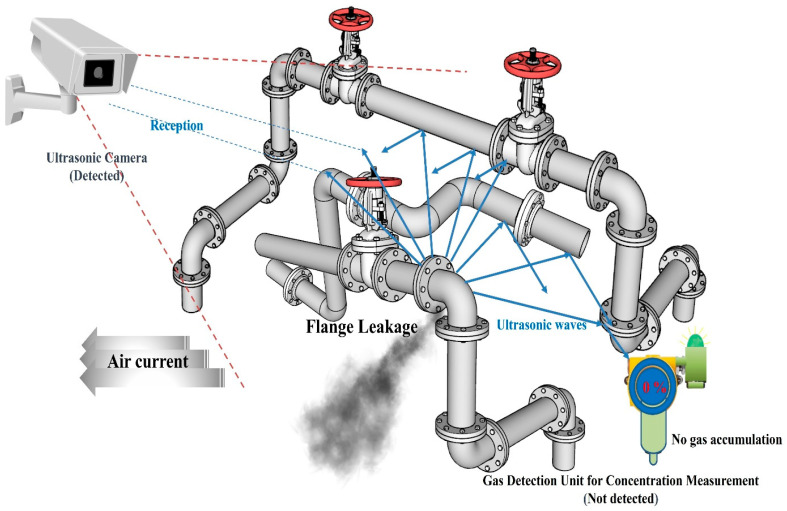
Concentration-measuring gas detector cannot be detected due to air current.

Figure 12 shows a case where only the positions of the ultrasonic sensor and concentration-measuring gas detector were changed under the same environmental conditions as Figure 11. In this case, the air flow was directed in the opposite direction of the concentration-measuring gas detector, so it could not accumulate at the location where the concentration-measuring gas detector was installed. However, the ultrasonic sensor received ultrasonic waves generated from the leak and detected the gas leak.

**Figure 13:**
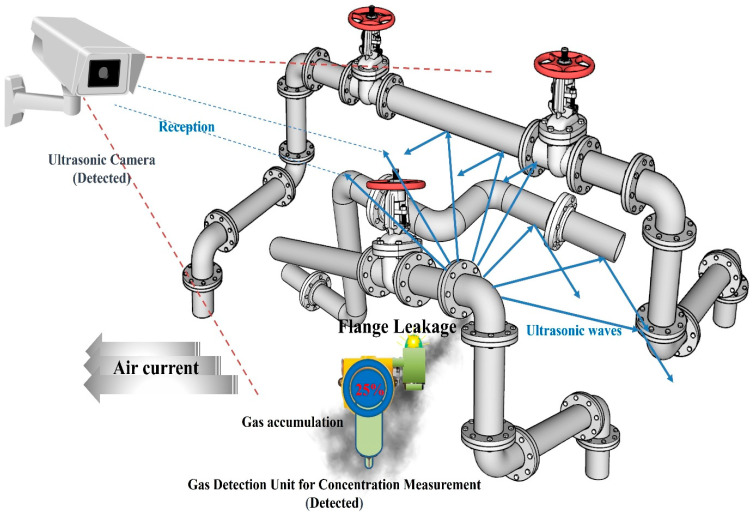
Optimal placement of ultrasonic cameras and gas leak detection alarms.

Figure 13 shows a case where the concentration-measuring gas detector is placed as shown in Figure 11 and the ultrasonic sensor is placed as shown in Figure 12 under the same environmental conditions as Figure 11 and Figure 12. In this case, the air flow was directed toward the concentration-measuring gas detector and accumulated, making it possible to detect gas leaks. An ultrasonic sensor was installed in a location that could receive ultrasonic waves generated from the leak, so the ultrasonic sensor was also able to detect gas leaks. As can be seen from the detection results in Figure 13, gas leak detection alarms are placed considering the airflow in the area. Ultrasonic sensors should be placed in a location where they can observe as much of the expected leak area as possible to minimize blind spots.

The integration of the two technologies can develop into a complementary relationship, minimizing blind spots in gas leak detection. Gas leaks can be detected more accurately and quickly, and reliability can be increased. Early response is possible with the introduction of an integrated technology that can accurately and quickly detect gas leaks, a major cause of accidents at petrochemical plants. Therefore, risks due to gas leaks can be reduced, and the safety of the workplace can be improved. The addition of an ultrasonic detection system can be considered while maintaining the existing system without having to consider demolition. A combination of the two systems can be proposed by placing an additional ultrasonic detection system in the blind spot of the existing gas leak detection system.

## 6. Discussion

Existing concentration-measuring gas leak detection systems installed in petrochemical plants are often located adjacent to facilities with a high risk of leakage, according to Korea’s “Technical Guidelines for the Installation and Maintenance of Gas Leak Detection Systems” (Kosha Guide P-166-2020) [36].

Therefore, one or more gas leak detection systems are deployed. Furthermore, Kosha Guide P-166-2020, Section 4.2, “Gas Leak Detection System Deployment Standards,” stipulates that at least one gas leak detection system be installed around key facilities, every 10 or 20 m around the floor surface. Applying this standard, at least one ultrasonic detection system per 20 m can be deployed. Ultrasound is a form of sound, and the travel time of ultrasound in air at 20 °C is known to be 343 m/s [42,43]. Therefore, it takes approximately 0.0583 s for sound to travel at a speed of 343 m/s to the microphone sensor of an ultrasonic camera located approximately 20 m away from the leak source. This means that a leak appears to be immediately detected by a human observer. However, currently developed fixed, continuous monitoring systems have a delay of approximately 1–3 s due to the camera’s cable and hub connection to the monitor, along with video processing time. This adds up to a total time of approximately 3.0583 s.

Previous studies have defined existing detection systems as approximately 9 s [37]. Therefore, it is expected that this system will not only shorten the detection time by one-third but also dramatically improve detection efficiency by minimizing missed detections.

Most existing gas leak detectors installed in petrochemical plants are chemical sensors that detect the concentration of gas leaked within a given space. It is difficult to detect gas concentrations that spread widely outdoors, and they cannot be detected if wind prevents the gas from reaching the gas sensor. Acoustic-based leak detection using conventional ultrasonic sensors can complement existing gas detection technologies.

Ultrasonic cameras differ from conventional sensors in that they utilize arrays of sensors, increasing sensitivity and enabling precise visualization of leak locations. This allows for rapid detection and prompt action. However, existing technologies require additional detection of leak locations, so methods such as spraying soap bubbles at all pipe joints are still used. Manually locating leaks increases the risk of human error and delays.

Furthermore, lower-frequency acoustic signals travel farther due to longer wavelengths, while higher-frequency sound waves are more susceptible to attenuation in air. Therefore, installing sensors to monitor areas where people have difficulty hearing from a distance and leaks may occur will be a way to reduce the manpower required for safety inspections and manage them effectively.

Through this study, gas leaks occurring in petrochemical complexes can be detected at an early stage to prevent accidents and reduce risks. Gas leak simulations can help identify the optimal position of existing gas leak detection systems. In a petrochemical plant, it is expected that the detection probability can be further increased if a leak detector is installed, considering the high probability of air flow directed toward the leak source.

In addition, more innovative safety management is possible by applying the recently developed ultrasonic detection technology. Ultrasonic detection can be used to detect ultrasonic waves generated when a leak occurs; therefore, it is less affected by air currents and can detect gas leaks at an early stage. The probability of detecting gas leaks at an early stage can be improved if gas leak detection is performed using ultrasonic sensors at locations where existing gas leak detection devices have difficulties measuring the gas concentration. Conversely, positioning an existing gas leak detection sensor within the blind spot of an ultrasonic detection sensor may be an effective method.

This methodological approach utilizing Windrose is specific to Korea and may have limitations based on a specific year. However, wind direction and speed are determined only by existing gas detectors and have no effect on ultrasonic detection. Therefore, ultrasonic detection can be applied consistently worldwide, regardless of the climate.

The proposed method is expected not only to improve the safety of gas leaks in petrochemical complexes but also to be applicable to gas-related facilities in other industries that are installed outdoors. Since simulations were used as a tool to verify the adequacy of detection, they have the limitation of simplifying complex environments when running Breeze simulations. We plan to further refine the system by identifying blind spots through more accurate simulations in the future. However, as each industrial site has its own characteristics, a simulation suitable for the business environment must be conducted. It should be noted that since the characteristics of each plant and process are different, the method proposed in this paper should be presented as an outcome that considers the characteristics of each location. Large workplaces, such as petrochemical plants, must pursue efficient approaches, including prioritizing high-risk areas.

## 7. Conclusions

In this study, we identified explosion-related risks using explosion simulations and explored methods to enhance the efficiency of existing gas leak detection systems using simulated leak scenarios. Finally, we incorporated the latest technology and reviewed the optimal arrangements of the existing gas detection and ultrasonic detection systems. Additionally, we proposed a method for minimizing the blind spots of gas leaks within the selected risk space.

(1) Simulation was performed using the BREEZE program. The material was assumed to be 1-butene, and the simulation results for each leak were confirmed. The estimation of the extent of damage from an explosion highlights the importance of gas leak detection.
(2) The gas distribution by airflow was predicted through a gas leak simulation, and a gas leak detector placement suitable for the area was proposed. It is predicted that the relocation of existing detection facilities can also increase the probability of gas leak detection. Considering wind direction and speed, the placement of efficient concentration-measuring gas leak detectors should be considered.
(3) Simulation results based on wind direction and speed associated with existing gas leak systems can vary by season and region. However, ultrasonic detection systems, which rely on visual observation of ultrasound waves, exhibit very little seasonal variation.
(4) Compared to existing gas leak systems and ultrasonic detection technology, detection time can be shortened by approximately one-third. Furthermore, existing and ultrasonic technologies have the characteristic of coexistence.
(5) An installation methodology combining an existing gas leak system and ultrasonic detection technology was presented. These two technologies work together to compensate for each other’s shortcomings. These suggestions for minimizing blind spots in leak detection can be used as effective countermeasures to mitigate damage by facilitating early detection and response to gas leaks.

## Figures and Tables

**Table 1:** Summary of explosions caused by gas leaks.

No.	Year	Number of Fires	Loss of Life			Property Damage Estimates (USD)
			Dead	Injury	Total	
1	2024	141	4	97	101	3,656,322
2	2023	130	2	98	100	1,525,585
3	2022	151	6	141	147	19,981,654
4	2021	146	1	78	79	2,001,183
5	2020	141	11	83	94	3,903,921
6	2019	162	7	117	124	4,214,131
7	2018	211	5	128	133	1,932,828

**Table 2:** Accident scenario element settings.

No.	Elements	Specifications
1	Equipment	Gas stripper (gas–liquid separation)
2	Temperature	120 °C
3	Pressure	16 bar
4	Height of stripper	13.7 m
5	AGL (above ground level to stripper bottom)	3.5 m
6	Height of leak source	17.2 m
7	Process piping (carbon steel pipe)	4-in inner diameter

**Table 3:** Parameter settings (2022).

Point	Temperature	Humidity	Surface Roughness Length	Wind Speed & Atmospheric Stability	Atmospheric Pressure
Yeosu Industrial Complex	15.4 °C	61%	High crops; scattered large obstacles (0.25 m)	1.5 m/s, F (very stable)	1.0163 bar

**Table 4:** Overpressure damage threshold.

Overpressure	Damage
0.07 bar	Window glass shatters. Light injuries from fragments occur.
0.21 bar	Residential structures collapse. Serious injuries are common, fatalities may occur.
0.84 bar	Probable total destruction of most buildings

**Table 5:** Scenarios of 1-butene explosion accident analysis.

Input Variable		Scenario 1	Scenario 2	Scenario 3
Type		VCE		
Confined volume (fuel-air)		0.5	1.0	1.5
Blast strength		7		
Results of overpressure	0.07 bar	10.0 m	12.6 m	14.4 m
	0.21 bar	4.8 m	6.0 m	6.9 m
	0.84 bar	1.6 m	2.0 m	2.3 m

**Table 6:** Concentration threshold.

Concentration	Explanation
0.4%	25% LEL of 1-butene
0.8%	50% LEL of 1-butene
1.6%	LEL of 1-butene

**Table 7:** Scenarios of 1-butene leakage accident analysis.

Input Variable		Scenario 1	Scenario 2
Wind speed		1.8 m/s	5.67 m/s
Wind direction		N (0°)	WNW (292.5°)
Atmospheric stability		F (Very stable)	
chemical stored		Compressed gas	
Release type		Horizontal jet (Continuous)	
Release hole		4 inches (Pipe diameter)	
Specify a duration		10 min	
Height of interest set to		17.2 m (same height as leak point)	
Results of concentration	0.4%	76.3 m	107.7 m
	0.8%	68.2 m	85.2 m
	1.6%	49.0 m	-

## Data Availability

Data available upon reasonable request with permission from the corresponding author and data management institution.
